# Modification of Branched Polyethyleneimine Using Mesquite Gum for Its Improved Hemocompatibility

**DOI:** 10.3390/polym13162766

**Published:** 2021-08-17

**Authors:** Ana M. Pinilla-Torres, Paola Y. Carrión-García, Celia N. Sánchez-Domínguez, Hugo Gallardo-Blanco, Margarita Sánchez-Domínguez

**Affiliations:** 1 Grupo de Química Coloidal e Interfacial Aplicada a Nanomateriales y Formulaciones, Centro de Investigación en Materiales Avanzados, S.C. (CIMAV, S.C.), Unidad Monterrey, Apodaca 66628, Mexico; ana.pinilla@cimav.edu.mx; 2 Departamento de Bioquímica y Medicina Molecular, Facultad de Medicina, Universidad Autónoma de Nuevo León, Monterrey 64460, Mexico; carriongarcia.paola@gmail.com (P.Y.C.-G.); celianohemi@hotmail.com (C.N.S.-D.); 3 Departamento de Genética, Facultad de Medicina, Universidad Autónoma de Nuevo León, Monterrey 64460, Mexico

**Keywords:** biocompatibility, branched polyethyleneimine, buffering capacity, functionalization, hemocompatibility, mesquite gum

## Abstract

In the present study, the modification of branched polyethyleneimine (b-PEI) was carried out using mesquite gum (MG) to improve its hemocompatibility to be used in biomedical applications. In the copolymer synthesis process (carboxymethylated mesquite gum grafted polyethyleneimine copolymer (CBX-MG-PEI), an MG carboxymethylation reaction was initially carried out (carboxymethylated mesquite gum (CBX-MG). Subsequently, the functionalization between CBX-MG and b-PEI was carried out using 1-ethyl-3-(3-dimethylaminopropyl) carbodiimide (EDC) and *N*-hydroxysuccinimide (NHS) as crosslinking agents. The synthesis products were characterized using Fourier transform infrared spectroscopy (FTIR), X-ray photoelectron spectroscopy (XPS), and thermogravimetric analysis (TGA). Thermogravimetric analysis showed that CBX-MG and CBX-MG-PEI presented a lower decomposition temperature than MG. The CBX-MG-PEI has a high buffer capacity in the pH range of 4 to 7, similar to the b-PEI. In addition, the CBX-MG-PEI showed an improvement in hemocompatibility in comparison with the b-PEI. The results showed a non-hemolytic property at doses lower than 0.1 µg/mL (CBX-MG-PEI). These results allow us to propose that this copolymer be used in transfection, polymeric nanoparticles, and biomaterials due to its physicochemical and hemocompatibility properties.

## 1. Introduction

Branched polyethyleneimine (b-PEI) is one of the most widely used synthetic polycations because of its chemical functionality arising from the high density of amines [1]. This polymer is obtained through cationic polymerization of aziridine, and it contains primary, secondary, and tertiary amine groups [2]. B-PEI has many potential applications due to its polycationic character and water solubility [3,4,5]. In biomedical applications, it has been used as an additive to improve the specificity and efficiency of the polymerase chain reaction (PCR) for DNA amplification [6]; enzyme immobilization [7,8]; construction of biosensors [9,10]; drug delivery [11,12] and gene therapy [13,14]. For the last application, b-PEI with a molecular weight of 25 kDa has been reported as one of the polymers most used as a non-viral vector for the transport of nucleic acids due to its ability to form polyplexes with nucleic acids through electrostatic interactions between the amino groups of b-PEI and the phosphate groups of nucleic acids [15,16,17]. Additionally, this polymer has shown high transfection efficiencies and a high buffer capacity. The latter property is associated with the ability of this polymer to facilitate the endosomal escape of nucleic acids (“proton sponge effect”) [18,19].

Although b-PEI has shown to be an excellent transfection vector, it has some limitations, among which are its cytotoxicity, low biodegradability, and low specificity, which gives rise to non-specific interactions with normal cells or blood components at the in vivo level, and this causes a decrease in the half-life of the *PEI*/*DNA* polyplexes in the bloodstream [20]. In order to overcome these limitations, some studies have reported the modification of b-PEI through functionalization with synthetic and natural polymers [21], for example, polysaccharides [22,23,24], poly (ethylene glycol) (PEG), poly (*ε*-caprolactone), and poly (l-lactide) (PLLA). In the case of polysaccharides, they have attracted the interest of many researchers in gene therapy due to their advantages, such as biocompatibility, biodegradability, low toxicity, and various modification strategies. Among the polysaccharides that have been used to carry out this modification are chitosan, cellulose, and natural gums [22,24,25]. Several strategies have been adopted to reduce polyethyleneimine (PEI) toxicity as the nucleic acid carrier [26,27]. PEI capacity as a nucleic acid carrier depends on the molecular weight, linear or branched molecular structure, chemical modifications, and combinations with molecules of biological or synthetic origin. Because of these variable factors, each nanoformulation needs to be characterized individually to evaluate their cytotoxicity, hemotoxicity, biocompatibility, and buffering properties. Additionally, the type of nucleic acid, molecular weight, and conformation contribute to modifying the factors previously mentioned factors.

However, there are not many studies about the use of natural gums modified with b-PEI. Among the reported studies is Goyal et al., who modified the PEI using gellan gum, a linear anionic heteropolysaccharide produced by a microorganism (Sphingomonas elodea). Its structure is based on a repeating tetrasaccharide unit composed of (1-3)-β-D-glucose, (1-4)-β-D-glucuronic acid, (1-4)-β-D-glucose, and (1-4)-α-L-rhamnose. The synthesized copolymer was biocompatible (in vitro studies, primary keratinocytes, HEK293, HeLa, and HepG2 cells). Additionally, the copolymer protected the DNA from enzymatic cleavage, reducing the interaction with serum proteins [23]. Additional examples are the studies by Jana et al. and Bansal et al., which reported using guar gum; this is a polygalactomannan derived from the seeds of a leguminacea plant, *Cyamopsis tetragonolobus*. It is composed of mannose and galactose, and is referred to as a galactomannan. In the study by Jana et al., the authors reported that the copolymer was more biocompatible for the cells and the blood than b-PEI. Additionally, they reported that the copolymer improved the in vitro gene transfection efficiency concerning b-PEI [22]. On the other hand, Bansal et al. developed a novel series of gene carriers by conjugating depolymerized guar gum with low molecular weight branched PEI to deliver nucleic acids simultaneously to macrophages and hepatocytes. The authors reported that all polyplexes showed significantly higher transfection [28].

The genus *Prosopis* has about 44 species (spiny trees, 10 mesquite species, distributed around the southern United States and Mexico) [29]. It is widely distributed as a native or introduced species in at least 129 island countries and territories, and this includes the Caribbean islands (18) and mainland countries (19) in the Americas (excluding Canada, Suriname, and Guyana), 40 countries in Africa, 26 in Asia, 4 in Europe, 24 island countries in the Pacific, Atlantic, and Indian Oceans, and Australia [30]. In Mexico, there are around 10 species of *Prosopis*, *Prosopis* *juliflora* being the most abundant. In the northwestern state of Sonora, *Prosopis velutina* and *Prosopis pubescens* are found [31]. These species have easy dispersion, grow fast, and adapt to survive and grow in desert environments [30,32].

Mesquite gum (MG) is constituted by residues of L-arabinose, D-galactose, 4-O-Methyl-D-glucuronic acid, and L-rhamnose [33,34]. MG’s functional properties are closely related to its structure, which determines, for example, the solubility, viscosity, and emulsification capacity. Regarding the solubility of MG, it is highly soluble in an aqueous solution. It exhibits excellent solubility up to 50% concentration in aqueous media [34], and unlike other gums of natural origin, such as guar gum and gellan gum, it does not require heating for dissolution [35,36]. Additionally, MG is considered a polyelectrolyte. Upon dilution, MG molecules dissociate into a polyvalent macroion and a large number of counterions, giving rise to an electrostatic field whose nature and interactions will determine the MG conformation in the solution. The presence of electrolytes in an aqueous gum solution decreases the ionization of gum molecules. [34]. Another essential property of MG is viscosity. Its viscosity is lower even at high concentrations compared to the viscosity of gellan gum, guar gum, and gum arabic [36,37,38]. Goycoolea et al. reported that MG solutions at 20% (*w*/*w*) in 0.1 M NaCl, measured at 20 °C, possess an average viscosity of 8.7 mL g^−1^ and Newtonian behavior [34].

Additionally, MG contains protein (3–7%) formed mainly by hydroxyproline, serine, glycine, and valine [37]. The presence of protein in mesquite gum is mainly responsible for its emulsification capacity and, in aqueous solution, it can reduce the surface tension to act as a steric stabilizer [38]. This last property has been used to develop methods of “green” synthesis of metallic nanoparticles [39]; these methods have advantages, such as reliability, sustainability, and eco-friendliness [40].

This article reports b-PEI (25 kD) modification with carboxymethylated mesquite gum (CBX-MG) by carbodiimide chemistry. The copolymer was characterized through Fourier transform infrared spectroscopy (FTIR), X-ray photoelectron spectroscopy (XPS), and thermogravimetric analysis (TGA). Additionally, buffering capacity was determined using titration methods. Finally, hemocompatibility was evaluated using hemolysis assay. The present study is the first study reporting the modification of PEI through the conjugation with MG and the first report that characterizes MG’s hemocompatibility.

## 2. Materials and Methods

The Ethics in the Research Committee of the School of Medicine and Dr. José Eleuterio Gonzalez University Hospital of the Universidad Autónoma de Nuevo León reviewed and approved this methodology in September 2017, with the project identification code BI17-00001. Therefore, the human donor was treated according to ethical standards.

### 2.1. Materials

Mesquite gum samples from *Prosopis velutina* were collected manually in the form of exudate pearls in the Mexican state of Sonora by local suppliers, and a batch was purchased in a convenient local store (Mieles de Sonora, Hermosillo, Mexico) and purified in the laboratory.

### 2.2. Mesquite Gum Purification

The protocol described by Moreno-Trejo et al. was used for the MG purification [39]. First, the mesquite exudate pearls were selected and cleaned following the method described in previous works [41,42,43], followed by pulverization in a mortar. After that, the powder was dissolved in distilled water at room temperature for 24 h. Once that time had passed for hydration of the gum, the liquid was filtered with a Whatman no. 2 filter paper, and then the filtered solution was frozen for 15 h and lyophilized in a FreeZone (Labconco, Kansas City, MO, USA) for 26 h.

### 2.3. Carboxymethylated Mesquite Gum Synthesis

**Figure 1:**
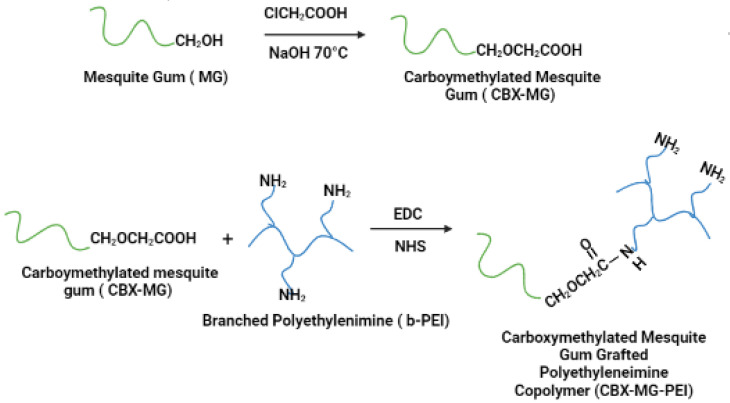
Schematic representation of carboxymethylation of mesquite gum and the modification of branched polyethyleneimine (b-PEI) with carboxymethylated mesquite gum (CBX-MG).

CBX-MG was synthesized by adapting the previously reported method by Niu et al. (Figure 1) [44]. First, 5 g of MG was weighed and dissolved in 70 mL of deionized water. Subsequently, 6 g of NaOH was weighed and dissolved in 30 mL of an aqueous ethanol solution (80% *v***/***v*). These two solutions were added to a three-neck balloon and mixed under constant stirring and at room temperature for 15 min. Then, 8 g of chloroacetic acid (Sigma-Aldrich, St. Louis, MO, USA) was added to the above mixture under stirring conditions, and the temperature was adjusted to 70 °C. The reaction was kept under the aforementioned conditions for 60 min. Finally, the heating and stirring were stopped, and 200 mL of cold ethanol was added to the solution.

### 2.4. Modification of b-PEI with CBX-MG

Carboxymethylated mesquite gum grafted polyethyleneimine copolymer (CBX-MG-PEI) was synthesized by adapting the previously reported method by Jana et al. (Figure 1) [22]. First, 3 g of CBX-MG was dissolved in 50 mL 0.1 M MES buffer (Sigma-Aldrich, St. Louis, MO, USA) to maintain the pH at 6.5. Then, 1-ethyl-3(3 dimethyl aminopropyl) carbodiimide (EDC)(Sigma-Aldrich, St. Louis, MO, USA) and *N*-hydroxysuccinimide (NHS) (Sigma-Aldrich, St. Louis, MO, USA) were added to the solution at a molar ratio of 1:4:4 (CBX-MG: EDC: NHS), and the reaction mixture was stirred at room temperature for 24 h to activate the carboxyl group of CBX-MG. Next, 15 g of b-PEI (25 kDa, Sigma-Aldrich, St. Louis, MO, USA) was dissolved in water, and it was added to the activated CBX-MG solution. Then, the pH of the solution was maintained at 6.5 and the reaction continued for another 48 h at room temperature. Finally, the resultant solution was purified by exhaustive dialysis against deionized water for 24 h, followed by lyophilization to obtain CBX-MG-PEI.

### 2.5. Characterization Techniques

FTIR analysis was carried out using a Thermo Nicolet 6700 FTIR (Thermo Fisher Scientific, Waltham, MD, USA). XPS analysis was carried out using an XPS Escalab 250Xi (Thermo Fisher Scientific, Waltham, MD, USA). Thermogravimetric analysis was carried out using a Q600 SDT thermal analyzer (TA Instruments, Leatherhead, UK) at a heating rate of 10 °C/min, ranging from room temperature to 800 °C, with a flow of nitrogen gas (purity 5.0) at 100 mL/min. The determination of potential zeta was carried out by a Zetasizer Nano ZS (Malvern Instruments, Malvern, UK). The absorbance measurements of the hemolysis tests were taken using the NanoDrop ND-1000 UV-Vis Spectrophotometer (Thermo Fisher Scientific, Waltham, MD, USA).

### 2.6. Determination of the Percentage of Conjugation of b-PEI with CBX-MG

#### 2.6.1. Semiquantitative Analysis through FTIR Spectroscopy

A semiquantitative analysis through FTIR spectroscopy was carried out. For this analysis, an appropriate baseline in the CBX-GM-PEI spectra was determined by using Origin software, and a Gaussian iterative curve fitting of the deconvoluted peaks afforded a means of evaluating the areas of each peak. The percentage of conjugation of b-PEI with CBX-MG was based on the peak area ratios of amide I/amine I band [45] and calculated through the following equation:

$$\%\;\mathrm{of}\;\mathrm{conjugation}\;\mathrm{bPEI}\;\mathrm{with}\;\mathrm{CBXMG}.=\frac{A_{\mathrm{amide}\;I}}{A_{\mathrm{amine}\;I}}\times 100$$

A_amide I:_ Area of the peak associated with amide I (1640.8 cm^−1^)

A_Amine I:_ Area of the peak associated with amine I (3420 cm^−1^)

#### 2.6.2. Semiquantitative Analysis through XPS Spectroscopy

A semiquantitative analysis through XPS spectroscopy was carried out. For this analysis, an appropriate baseline in the CBX-MG-PEI spectra was determined by using *Origin* software, and a Gaussian iterative curve fitting of the deconvoluted peaks afforded a means of evaluating the areas of each peak. The percentage of conjugation of b-PEI with CBX-MG was based on the peak area ratios of A_amide_/A_total area_ and calculated through the following equation:

$$\%\;\mathrm{of}\;\mathrm{conjugation}\;\mathrm{bPEI}\;\mathrm{with}\;\mathrm{CBXMG}.=\frac{A_{\mathrm{amide}}}{A_{\mathrm{total}\;\mathrm{area}}}\times 100$$

A_amide:_ Area of the peak associated with amide in the N1s spectra (399.8 cm^−1^)

A_total area:_ Sum of the areas in the N1s spectra

### 2.7. Buffering Capacity of CBX-MG-PEI

The CBX-MG-PEI polymer solution was prepared in a 50 mL flask (0.2 mg/mL, 30 mL), and pure water was used as a control. After adjusting the initial pH to 10.0 with 0.1 M NaOH, 25 µL increments of 0.1 M HCl were titrated into the solution while measuring the pH response with a pH electrode. The recorded pH varied from 10.0 to 3.0 [46].

### 2.8. Hemolysis Test

A hemolysis test was developed to determine the effect of polymers on erythrocyte lysis. The protocol followed in the present work was based on the investigation reported by Roacho et al. with some modifications [47].

A spray-coated EDTA tube (Becton Dickinson, Franklin Lakes, NJ, USA) with blood from a healthy donor was used. Erythrocytes were isolated by centrifugation (3000 rpm for 4 min) and washed three times with phosphate-buffered saline (PBS). Polymers were dispersed in PBS (0.6–0.01 µg/mL). Quadrupled samples of polymers were incubated with 98 µL of an erythrocytes suspension (1:99 erythrocytes:PBS). The incubation conditions were 37 °C in agitation at 300 rpm for 30 min. The positive control was a suspension of erythrocytes in distilled water. The negative control was a suspension of erythrocytes in PBS. After the incubation, the samples were centrifuged (14,000 rpm for 4 min), and the hemoglobin that was released in the supernatant was measured by ultraviolet–visible (UV–vis) spectroscopy at 415 nm. The positive control absorbance was a reference as 100% of hemolysis, and each of the samples was calculated according to the positive control. The average absorbance of the negative control was subtracted from each sample.

## 3. Results and Discussion

### 3.1. FTIR Spectra of the MG, CBX-MG, and CBX-MG-PEI

**Figure 2:**
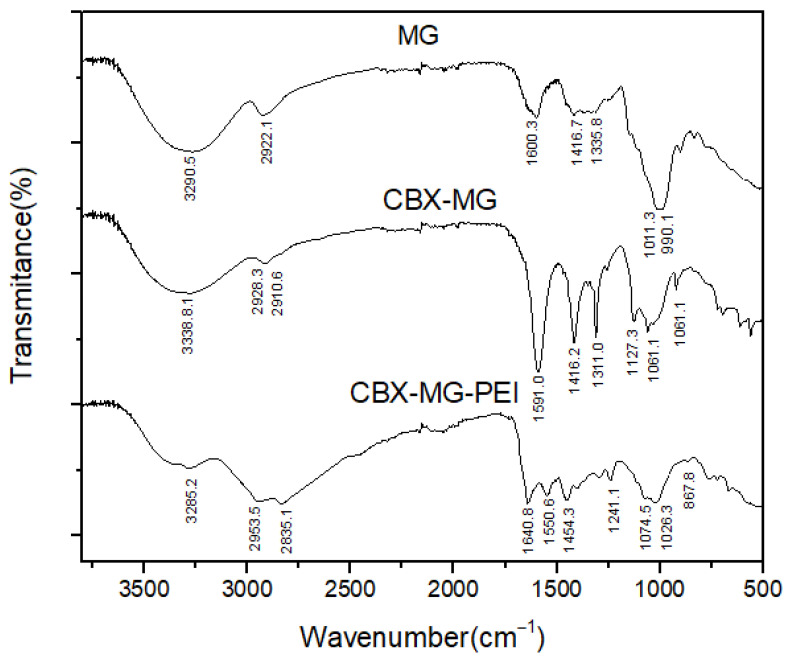
FTIR spectra of MG, CBX-MG and CBX-MG-PEI.

FTIR spectroscopy was used to further confirm the chemical modification of b-PEI with MG through carbodiimide chemistry (Figure 2). The FTIR spectra of the solid MG showed O–H and C–H bands at 3290 cm^−1^ and 2922 cm^−1^, respectively. A band centered near 1600 cm^−1^ is assigned to amide I, attributed to the protein content of the samples (arising from the MG glycoprotein); COO− asymmetric stretching bands, located at 1416 cm^−1^, were also identified. The bands around 1011 cm^−1^ and 990 cm^−1^ can be attributed to vibration modes of the C–O and the C–O–H groups of carbohydrates (such as glucose, mannose, and galactose). The band at 834 cm^−1^ indicates the occurrence of pyranose glycosidic acetal groups. According to Moreno-Trejo et al., these results were reported for the characterization of purified MG, and this material was used to synthesize silver nanoparticles and stabilize essential citrus oil nanoemulsions [39,48]. On the other hand, in the CBX-MG spectrum, three new bands appeared at 1591cm^−1^, 1310cm^−1^, and 1060 cm^−1^, corresponding to the COO− asymmetric and symmetric stretching vibration due to the carboxyl group of the carboxymethyl moiety of CBX-MG. These results correlate with those obtained by other authors who have carried out carboxymethylation in other polysaccharides [49,50,51].

Regarding the results obtained for the CBX-MG-PEI, peaks associated with the primary amine (3424 cm^−1^ and 3280 cm^−1^) and the amide group (3280 cm^−1^, 1640.8 cm^−1^, 1550.6 cm^−1^, 1241.1 cm^−1^, and 1074.5 cm^−1^) were evidenced. The assignments of these peaks can be observed in Table 1. The peaks associated with the amide group show that the reaction between the carboxyl groups of carboxymethylated CBX-MG and the amine groups present in b-PEI took place. These results confirmed that the CBX-MG-PEI copolymer was successfully synthesized.

### 3.2. XPS Spectra of the MG, CBX-MG, and CBX-MG-PEI

**Figure 3:**
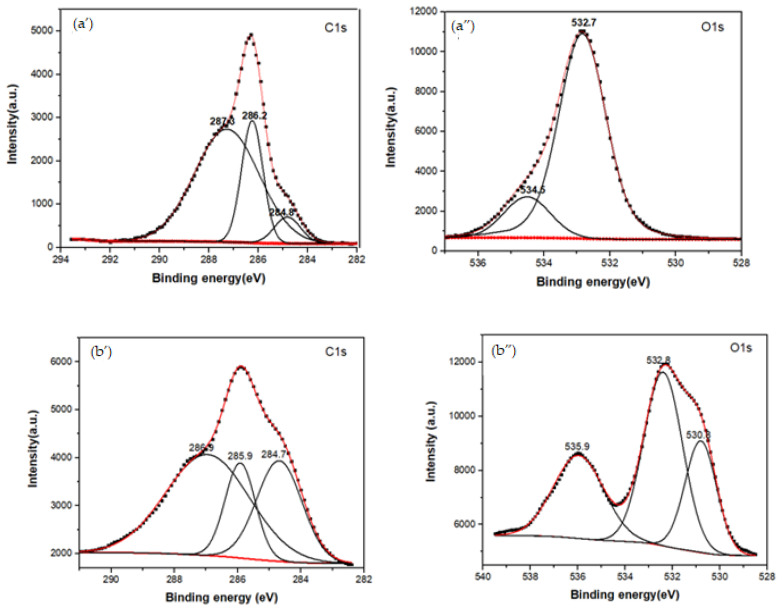

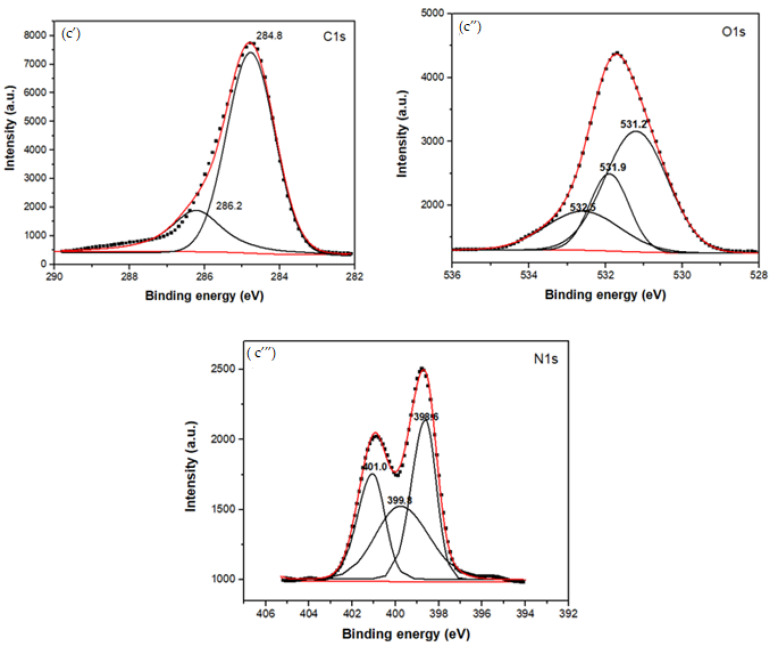
XPS spectra of the MG, CBX-MG, and CBX-MG-PEI: (**a**) XPS spectra of MG: (**a’**) Deconvolution of C1s peak (**a”**) Deconvolution of O1s peak; (**b**) XPS spectra of CBX-MG: (**b’**) Deconvolution of C1s peak (**b”**) Deconvolution of O1s peak (**c**) XPS spectra of CBX-PEI-MG: (**c’**) Deconvolution of C1s peak (**c”**) Deconvolution of O1s peak (**c’”**) Deconvolution of N1s peaks.

XPS spectroscopy was used to obtain information about the functionalization between b-PEI and CBX-MG at a superficial level. The elements selected to be analyzed by this technique were C1s, O1s, and N1s. Three peaks were deconvoluted in the C1s spectrum for the MG (Figure 3a’ and Table 2), which were assigned to the adventitious carbon (284.8 eV), C-OH (286.2 eV), and O-C=O (287.3 eV), respectively [52]. When comparing these results with those obtained for the CBX-MG, the appearance of a new signal in the O1S spectrum (Figure 3b” and Table 2) at 532.8 eV associated with the presence of ether groups (C-O-C) is evidence of MG carboxymethylation. Additionally, the signals in the C1s spectrum (Figure 3b’ and Table 2) for CBX-MG associated with C-OH/C=O had a chemical shift with respect to those obtained for the MG. These chemical shifts could be associated with changes in the oxidation state of the analyzed element. The results obtained are correlated with those reported by other authors who characterized carboxymethylated polysaccharides using this technique [52,53].

For the copolymer made up of CBX-MG and branched PEI (25 kD), the absence of the signal associated with the O-C=O group was evidenced in the C1s spectrum (Figure 3c’ and Table 2), which indeed appears in the spectra of MG and CBX-MG (287.3 and 286.9 eV, respectively). Additionally, the presence of a new band at 286.2 eV that can be associated with amide groups [54] was evidenced in the C1s spectrum (Figure 3c’ and Table 2). For the case of the O1s spectrum (Figure 3c” and Table 2), two bands associated with amide groups were evidenced. These bands were C=O (531.9 eV and O=C-N (531.2 eV) [52,53]. Finally, the deconvoluted three bands in the N1s spectrum for CBX-MG-PEI (Figure 3c’” and Table 2) were assigned to primary amines (398.6 eV), amide groups (399.8 eV), and protonated amines (401 eV) [53,54,55].

These results confirmed that the CBX-MG-PEI copolymer was successfully synthesized through the formation of the amide bond between carboxyl groups of carboxymethylated CBX-MG and the amino groups in b-PEI.

### 3.3. Determination of the Percentage of Conjugation of b-PEI with CBX-MG

**Figure 4:**
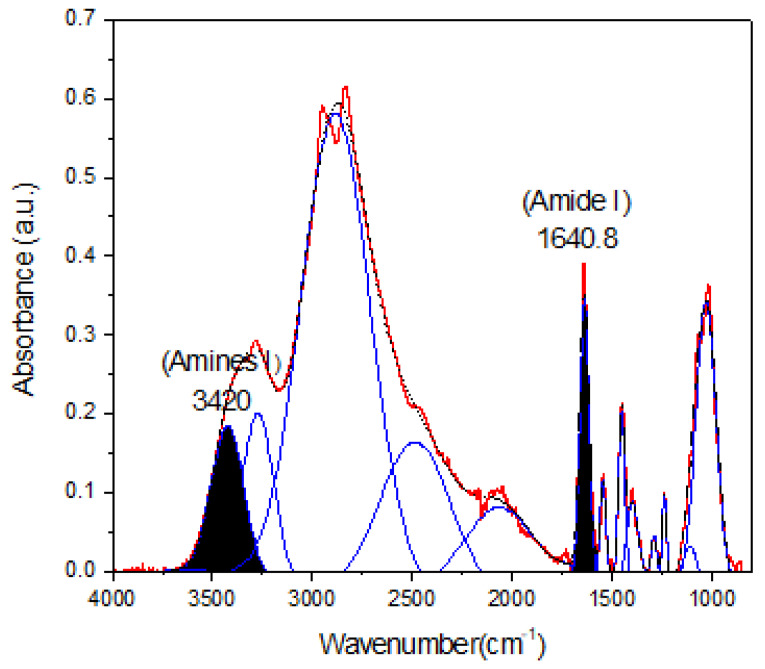
Deconvoluted spectra of CBX-MG-PEI. The peaks at 1640.8 cm^−1^ and 3420 cm^−1^ were taken as a reference to peak area ratios of amide I/amine I.

The percentage of conjugation of PEI with carboxymethylated mesquite gum was calculated through semiquantitative analysis through FTIR and XPS spectroscopy (N1s spectra) based on the calculation of the peak area ratios ((A_amide I_/A_amine_) [45] and A_amide_/A_total area_, respectively). The results obtained are shown in Figure 4 and Table 3 and Table 4.

Taking into account the results shown in Figure 4 and Table 3 and Table 4, the percentages of conjugation obtained through FTIR and XPS were 48.8% and 53%, respectively. These results could be correlated with the percentage obtained by Jana et al. through 2, 4,6-Trinitrobenzene Sulfonic Acid (TNBS) assay (43.22%) [22]. Additionally, comparison of the absorbance of the peaks corresponding to amine I and amide I showed an increase in absorbance for amide I as compared to that obtained for amine I. This increase is associated with a greater formation of amide bonds between the carboxyl groups of mesquite gum and the amino groups of b-PEI.

### 3.4. Thermogravimetric Analysis (TGA)

The TGA technique allows studying the decomposition pattern and thermal stability of materials. The results (Figure S1) and discussion are in the Supplementary Materials section.

### 3.5. Determination of Zeta Potential

The zeta potential is an important parameter to examine the surface charge of the polymers, depending on the pH of the solution. CBX-MG-PEI showed higher positive zeta potential in the acidic pH region due to the protonation of the amino groups of b-PEI. On the other hand, at the higher pH range, CBX-MG-PEI showed less positive zeta potential value due to fewer positively charged ions (Table 5). The results suggest that the amine groups on the surface of CBX-MG-PEI could be used to bind the negatively charged DNA molecules to form nanoparticle complexes (polyplexes) [22,24,56]. Additionally, positively charged polymers or nanoparticles are more susceptible to being bound and internalized into cancer cells [57], thus achieving better efficiency in the transfection process when used as vectors in gene therapy.

### 3.6. Buffering Capacity

**Figure 5:**
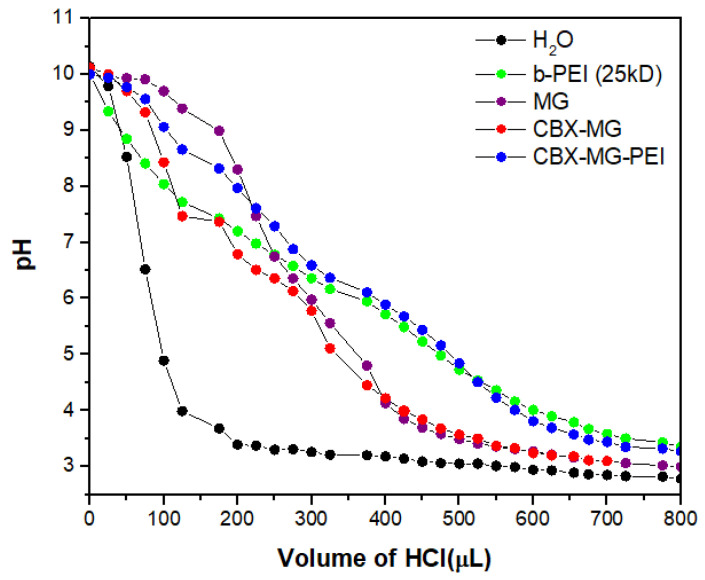
Determination of the buffering capacity of copolymer and precursors.

The majority of cationic polymers have a high buffering capacity, disrupting endosomes during transfection, thereby facilitating the escape of the polymer/DNA complex [46,58]. Figure 5 shows that the CBX-MG-PEI had a relatively high buffering capacity at a pH ranging from 4 to 7, compared with pure water. Additionally, its buffering capacity is similar to the capacity obtained for b-PEI (25 kD). The CBX-MG-PEI had a slightly higher buffering capacity than the b-PEI and this result can be related to the content of proteins in mesquite gum. Proteins behave as good buffers, due to their acid–base groups (amino and carboxylic groups), with the highest resistance to change in pH when the pH is close or equal to their pKa [59,60]. These results indicated that CBX-MG-PEI could be a potential non-viral gene vector.

### 3.7. Hemolysis Assay

**Figure 6:**
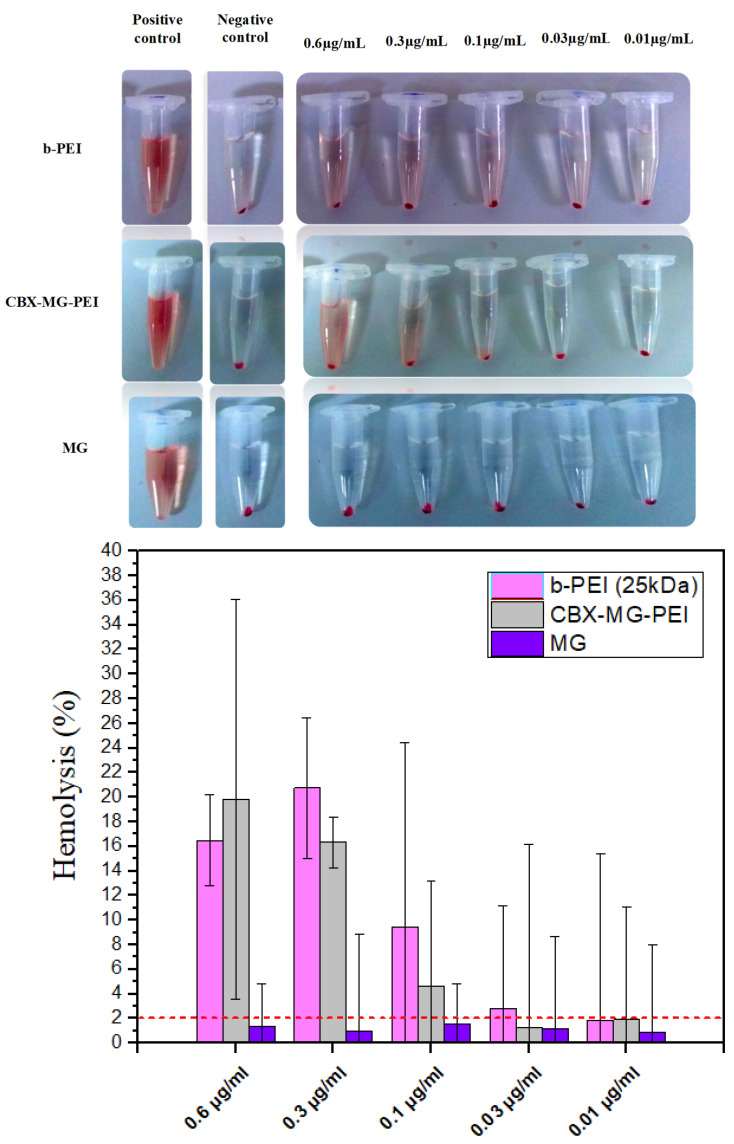
Hemolysis assay results obtained for b-PEI, CBX-MG-PEI, and MG.

A hemolysis assay is an essential initial step in evaluating the blood compatibility of polymers since this test can predict the potential side effects for polymeric vectors in intravenous administration [61]. This assay measures the lysis of the red blood cells exposed to an environmental agent. This lysis produces the release of the intracellular content of the erythrocyte due to the rupture of its membrane. The released molecule measured was hemoglobin, which is a predominant protein in erythrocytes [47]. To the best of our knowledge, there are few works that give a report on the hemocompatibility of natural gums, in the case of the work by Goyal et al. [23], they did not carry out hemolysis testing. On the other hand, Jana et al. [22] carried out a qualitative hemolysis test, but they did not calculate the percentages of hemolysis obtained. For this assay, concentrations from 0.6 to 0.01 µg/mL of the copolymer, mesquite gum, and polyethyleneimine were evaluated. Following the standard practices, the results for *Assessment of Hemolytic Properties of Materials ASTM F756-08* were analyzed, which indicate that the hemolytic activity of the materials is classified in three types: non-hemolytic materials (0–2% of hemolysis), low hemolytic materials (2–5% of hemolysis), and high hemolytic materials (higher than 5% [62]. For the case of b-PEI, it was found to induce hemolysis (Figure 6) due to its large molecular mass and high charge density resulting from a large number of secondary amine groups [63]. In contrast, CBX-MG-PEI had a hemolysis rate lower than 2% at 0.03 and 0.01 µg/mL, making it a non-hemolytic material at those concentrations (Figure 6). Additionally, the hemolysis rate of MG in concentrations between 0.01 and 0.6 µg/mL was found under 2% (Figure 6), in agreement with previously reported results where gum arabic presented hemolysis of 1.2 ± 0.2%, showing non-hemolytic activity [64]. The improved hemocompatibility of CBX-MG-PEI compared to b-PEI can be associated with the shielding from the positive charges of b-PEI due to the presence of MG. These results are correlated with those reported in the literature, which reports the modification of PEI with PEG and natural polymers [22,23,65].

## 4. Conclusions

This study developed a novel copolymer based on carboxymethylated mesquite gum grafted polyethyleneimine, characterized by FTIR, XPS, zeta potential, and TGA. The copolymer keeps an essential property of b-PEI, which is the buffer capacity. This property is critical for its application as a non-viral vector in gene therapy. Additionally, the results obtained in the hemolysis assays indicate that the introduction of MG in the structure of b-PEI decreases the hemotoxicity of the latter. This knowledge will open different copolymer applications, for example, as a non-viral vector in gene therapy against cancer. These results allow us to propose that this copolymer can be investigated in transfection studies, preparation of polymeric or metallic nanoparticles, and in general as a biomaterial due to its physicochemical and hemocompatibility properties.

## Figures and Tables

**Table 1:** Peak assignments associated with the formation of the amide bond in CBX-MG-PEI.

Peak Position (cm^−1^)	Peak Assignment
3420.0	Primary amines (N-H stretching)
3280.0	Amide A (N-H stretching), Primary amines (N-H stretching)
1640.8	Amide I (stretching of the C=O and C-N)
1550.6	Amide II (in-plane N-H bending, C-N and C-C stretching)
1241.1	Amide III (C-N stretching and N -H bending)
1074.5	C-N stretching

**Table 2:** XPS peak assignments of the MG, CBX-MG, and CBX-MG-PEI.

**MG**			
C1s		O1s	
Binding energy (eV)	Peak Assignment	Binding Energy (eV)	Peak Assignment
287.3	O-C=O	534.5	O-C=O
286.2	C-OH	532.7	C-OH
284.8	C-C/C-H		
**CBX-MG**			
C1s		O1s	
Binding energy (eV)	Peak Assignment	Binding Energy (eV)	Peak Assignment
286.9	O-C=O	530.8	C=O
285.9	C-OH	532.8	C-O-C
284.7	C-C/C-H	535.9	H_2_ O
**CBX-MG-PEI**			
C1s		O1s	
Binding energy (eV)	Peak Assignment	Binding Energy (eV)	Peak Assignment
286.2	C-N	532.5	C-O
284.8	C-C/C-H	531.9	C=O
		531.2	O=C-N
	N1s		
	Binding energy (eV)	Peak assignment	
	401.0	Protonated amines	
	399.8	Amide (O=C-N)	
	398.6	Primary amines	

**Table 3:** Determination of the areas of the peaks in the FTIR spectra of CBX-MG-PEI.

Peak Assignment	Wavenumber (cm^−1^)	Area
Amine I	3420.0	38.7
Amide I	1640.8	18.9

**Table 4:** Determination of the areas of the peaks in the XPS spectra of CBX-MG-PEI.

Peak Assignment	Binding Energy (eV)	Area
Protonated amines	398.6	2050.5
Amide	399.8	2436.4
Primary amines	401.0	110.0

**Table 5:** Measurements of potential zeta for CBX-MG-PEI at different pH.

pH	Zeta Potential (mV)
4	+45 ± 5.89
6.5	+39.1 ± 7.80
8	+31.4 ± 13.1

## Data Availability

The data presented in this study are available on request from the corresponding author.

## Supplementary Materials

The following are available online at https://www.mdpi.com/article/10.3390/polym13162766/s1, Figure S1. Thermogravimetric analysis of MG, CBX-MG, and CBX-MG-PEI.
