# Supplementary material for: Modification of Branched Polyethyleneimine Using Mesquite Gum for Its Improved Hemocompatibility
Source: Polymers (Basel). 2021 Aug 17;13(16):2766. doi: 10.3390/polym13162766 (PMC8399277; doi:10.3390/polym13162766)
Supplement: Supplementary file 1 [file polymers-13-02766-s001.zip › polymers-1273581-supplementary.pdf]

Supplementary Materials

Modification of Branched Polyethyleneimine Using Mesquite Gum for its Improved Hemocompatibility

Ana M. Pinilla-Torres, Paola Y. Carrión-García, Celia N. Sánchez-Domínguez, Hugo Gallardo-Blanco and Margarita Sánchez-Domínguez

Thermogravimetric analysis (TGA)

The TGA technique allows studying the decomposition pattern and thermal stability of the materials. Figure S1 shows the TGA graphs obtained for MG, CBX-MG, and CBX-MG-PEI.

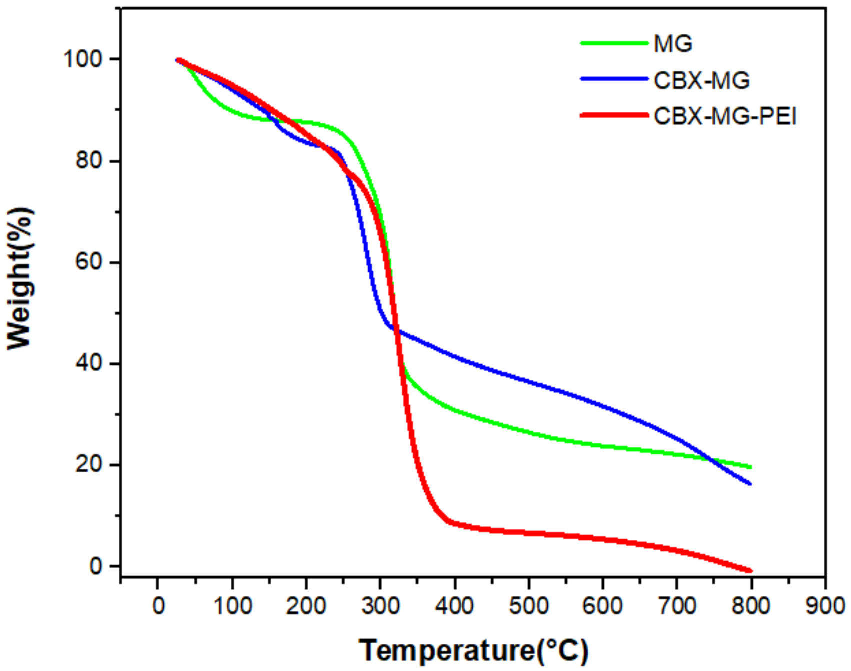

Figure S1. Thermogravimetric analysis of MG, CBX-MG and CBX-MG-PEI.

The TGA graph of the MG shows that two stages govern the decomposition of the polymer. The first stage of weight loss (range 60 °C-100 °C) is assumed to be due to moisture contained in the samples. On the other hand, the second stage is related to the decomposition process characteristic of the polysaccharides that make up the gums. This second stage begins at 200 °C [1–2].

Comparing the three TGA graphs it is shown that the samples of CBX-MG and CBX-MG-PEI have a lower decomposition temperature than MG. This decrease in thermal stability can probably be attributed to the breakdown of the covalent interaction, between the substituent and the polysaccharide [3–5].

Among the three polymers, the one considered to have the lowest thermal stability is CBX-MG-PEI; this was evidenced by the percentage of weight loss in the decomposition stage (79.7%). The sample under high-temperature conditions is degraded. A contrary result occurs in the MG and CBX-MG samples, which after the decomposition process

at high temperatures, they are left with a residue of 20 and 17%, respectively.

References

1. Moreno-Trejo, M.B.; Sánchez-Domínguez, M. Mesquite Gum as a Novel Reducing and Stabilizing Agent for Modified Tollens Synthesis of Highly Concentrated Ag Nanoparticles. *Mater.* **2016**, *9*, 817, doi:10.3390/ma9100817.
2. Hongbo, T.; Yanping, L.; Min, S.; Xiguang, W. Preparation and property of crosslinking guar gum. *Polym. J.* **2011**, *44*, 211–216, doi:10.1038/pj.2011.117.
3. Pettignano, A.; Charlot, A.; Fleury, E. Solvent-Free Synthesis of Amidated Carboxymethyl Cellulose Derivatives: Effect on the Thermal Properties. *Polym.* **2019**, *11*, 1227, doi:10.3390/polym11071227.
4. Kaity, S.; Ghosh, A. Carboxymethylation of Locust Bean Gum: Application in Interpenetrating Polymer Network Microspheres for Controlled Drug Delivery. *Ind. Eng. Chem. Res.* **2013**, *52*, 10033–10045, doi:10.1021/ie400445h.
5. Santos, M.B.; dos Santos, C.H.C.; de Carvalho, M.G.; Carvalho, C.W.P.; Garcia-Rojas, E.E. Physicochemical, thermal and rheological properties of synthesized carboxymethyl tara gum (Caesalpinia spinosa). *Int. J. Biol. Macromol.* **2019**, *134*, 595–603, doi:10.1016/j.ijbiomac.2019.05.025.
